# Prolonged tuberculosis-associated immune reconstitution inflammatory syndrome: characteristics and risk factors

**DOI:** 10.1186/s12879-016-1850-2

**Published:** 2016-09-27

**Authors:** Tasnim M. Bana, Maia Lesosky, Dominique J. Pepper, Helen van der Plas, Charlotte Schutz, Rene Goliath, Chelsea Morroni, Marc Mendelson, Gary Maartens, Robert J. Wilkinson, Graeme Meintjes

**Affiliations:** 1 Department of Medicine, University of Cape Town, Observatory, 7925 South Africa; 2 Critical Care Medicine Department, National Institutes of Health, Bethesda, MD USA; 3 Division of Infectious Diseases and HIV Medicine, Department of Medicine, University of Cape Town, Observatory, 7925 South Africa; 4 Clinical Infectious Diseases Research Initiative, Institute of Infectious Disease and Molecular Medicine, Faculty of Health Sciences, University of Cape Town, Anzio Road, Observatory, 7925 South Africa; 5 Institute for Women’s Health and Institute for Global Health, University College London, London, UK; 6 Division of Clinical Pharmacology, Department of Medicine, University of Cape Town, Observatory, 7925 South Africa; 7 Francis Crick Institute, London, NW7 1AA UK; 8 Department of Medicine, Imperial College London, London, W2 1PG UK

**Keywords:** Tuberculosis, HIV, Immune reconstitution inflammatory syndrome, IRIS, Glucocorticoids

## Abstract

**Background:**

In a proportion of patients with HIV-associated tuberculosis who develop paradoxical immune reconstitution inflammatory syndrome (IRIS), the clinical course of IRIS is prolonged necessitating substantial health care utilization for diagnostic and therapeutic interventions. Prolonged TB-IRIS has not been prospectively studied to date. We aimed to determine the proportion of patients with prolonged TB-IRIS, as well as the clinical characteristics and risk factors for prolonged TB-IRIS.

**Methods:**

We pooled data from two prospective observational studies and a randomized controlled trial conducted in Cape Town, South Africa, that enrolled patients with paradoxical TB-IRIS. We used the same diagnostic approach and clinical case definitions for TB-IRIS in the 3 studies. Prolonged TB-IRIS was defined as TB-IRIS symptoms lasting > 90 days. Risk factors for prolonged TB-IRIS were analysed using Wilcoxon rank sum test, Fisher’s exact test, multivariate logistic regression and Cox proportional hazards models.

**Results:**

Two-hundred and sixteen patients with TB-IRIS were included. The median duration of TB-IRIS symptoms was 71.0 days (IQR 41.0–113.2). In 73/181 patients (40.3 %) with adequate follow-up data, IRIS duration was > 90 days. Six patients (3.3 %), mainly with lymph node involvement, had IRIS duration > 1 year. In univariate logistic regression analysis the following were significantly associated with IRIS duration > 90 days: lymph node involvement at initial TB diagnosis, drug-resistant TB, lymph node TB-IRIS, and not being hospitalised at time of TB-IRIS diagnosis. In our multivariate logistic regression model lymph node TB-IRIS (aOR 2.27, 95 % CI 1.13–4.59) and not being hospitalised at time of TB-IRIS diagnosis (aOR for being hospitalised 0.5, 95 % CI 0.25-0.99) remained significantly associated with prolonged TB-IRIS, and drug-resistant TB was of borderline significance (aOR 3.26, 95 % CI 0.97–12.99). The association of not being hospitalised with longer duration of IRIS might be related to 1 of the 3 cohorts in which all patients were hospitalised at ART initiation with close inpatient follow-up. This could have resulted in diagnosis of milder cases and earlier IRIS treatment potentially resulting in shorter TB-IRIS duration in these hospitalised patients.

**Conclusions:**

Around 40 % of patients with TB-IRIS have symptoms for more than 90 days. Involvement of lymph nodes at time of TB-IRIS is an independent risk factor for prolonged TB-IRIS. Future studies should address whether more prompt anti-inflammatory treatment of lymph node TB-IRIS reduces the risk of prolonged TB-IRIS.

**Trial registration:**

The randomized controlled trial was registered with Current Controlled Trials ISRCTN21322548 on 17 August 2005.

**Electronic supplementary material:**

The online version of this article (doi:10.1186/s12879-016-1850-2) contains supplementary material, which is available to authorized users.

## Background

Paradoxical tuberculosis-associated immune reconstitution inflammatory syndrome (TB-IRIS) is an immunopathological reaction occurring in 4–54 % patients who start antiretroviral therapy (ART) while on treatment for tuberculosis (TB) [1–3]. Mortality directly attributed to paradoxical TB-IRIS is not frequent [4]. However, TB-IRIS causes substantial morbidity, necessitating hospitalisation and health care utilisation for diagnostic and therapeutic procedures [5, 6], particularly when TB-IRIS has a protracted clinical course. The median duration of TB-IRIS symptoms reported from several observational studies and clinical trials has been 1–3 months [5–11]. However, the condition may persist longer, more than 1 year in certain cases [2, 5, 9–12]. Prolonged corticosteroid therapy in such patients may be associated with significant complications.

The risk factors and clinical features of patients who develop a prolonged course of TB-IRIS have not been systematically studied. We conducted this study to determine the proportion of TB-IRIS patients that experienced a prolonged clinical course (defined as symptoms lasting > 90 days), as well as their clinical characteristics, outcomes, and risk factors.

## Methods

### Study design

We pooled data from three prospective studies of TB-IRIS that our group conducted in Cape Town, South Africa between 2005 and 2010 [13–15]. Data from eligible TB-IRIS cases assessed and followed up in these studies were included in this combined *post hoc* analysis. One of these studies was a randomized controlled trial (RCT) and the other two were prospective observational cohort studies. The RCT (*n* = 110) was conducted at GF Jooste Hospital with enrollment between June 2005 and December 2007. This was a secondary-level university-affiliated hospital serving communities with high HIV/TB co-infection rates. Patients were usually started on TB treatment and ART in primary care clinics and referred to the hospital with suspected TB-IRIS. The treatment intervention was a 4-week course of prednisone (1.5 mg/kg/day for 2 weeks followed by 0.75 mg/kg/day for a further 2 weeks). The trial was placebo-controlled with 1:1 randomisation. Trial design allowed patients who deteriorated or relapsed after stopping study drug to be commenced on open-label prednisone at clinician discretion. The trial duration was 12 weeks, but if patients still had TB-IRIS symptoms at 12 weeks follow-up was continued until TB-IRIS resolved [14].

The first observational cohort study (Cohort 1) was also conducted at GF Jooste Hospital at the same time as the RCT [13]. Between February 2005 and July 2006, 61 patients with TB-IRIS who were seen prior to the RCT or were ineligible for the RCT (for example because of neurological TB-IRIS) were enrolled. These patients were followed as outpatients until resolution of TB-IRIS symptoms. The second observational cohort study (Cohort 2) was conducted at Brooklyn Chest TB Hospital, also in Cape Town, with recruitment between May 2009 and November 2010 [15]. This was a study of ART-naïve patients who required hospitalisation for the treatment for HIV-associated TB and who started ART while admitted to this TB hospital and were followed for at least 12 weeks. Forty-seven TB-IRIS cases were diagnosed in this study and patients were followed for longer than 12 weeks if TB-IRIS was ongoing.

TB was diagnosed microbiologically (smear or culture) or using World Health Organization (WHO) guidelines for the diagnosis of smear-negative pulmonary and extrapulmonary TB [16]. Patients were treated according to national guidelines for TB and HIV. During the study periods TB cases received 6 months of therapy (rifampicin, isoniazid, pyrazinamide, and ethambutol for 2 months, followed by rifampin and isoniazid for 4 months). The retreatment regimen included the addition of streptomycin for the first 2 months of an 8-month regimen. Routine TB drug susceptibility testing (DST) was not performed for new TB cases. Patients receiving retreatment and patients not responding to TB treatment may have had DST performed. DST was only performed for rifampin, isoniazid, and ethambutol using culture-based techniques as previously described [13]. When multi- drug resistant TB was diagnosed a standard multi-drug resistant TB regimen was prescribed. First-line ART in South Africa at the time of these studies was stavudine, lamivudine, and either nevirapine or efavirenz. Efavirenz was preferred for patients receiving rifampicin-based TB treatment. Patients with a CD4 cell count <200 cells/μl and/or World Health Organization stage 4 disease were eligible to commence ART. In April 2010, the guidelines changed: tenofovir replaced stavudine, and TB patients were eligible for ART with a CD4 count < 350 cells/μl. Laboratory tests were performed by the National Health Laboratory Services (NHLS).

The same approach to diagnosing TB-IRIS was used across the studies. In those with suspected TB-IRIS, our clinical assessment sought to exclude differential diagnoses based on clinical presentation (for example, in those with respiratory symptoms, bacterial and pneumocystis pneumonia were investigated). In TB-IRIS patients, sputum (or extra-pulmonary) samples were sent when they could be obtained to exclude rifampicin resistant TB.

### Study definitions

All patients included in this analysis fulfilled the International Network for the Study of HIV-associated IRIS (INSHI) consensus case definition for paradoxical TB-IRIS [2] with one exception: not all patients with drug resistant TB were excluded. We have previously described an overlap of TB-IRIS and rifampicin resistant TB [13] and for this reason when a patient with drug resistant TB was also considered to have TB-IRIS they were not excluded from the analyses as we wished to determine if underlying drug resistant TB was a risk factor for prolonged TB-IRIS.

Pulmonary TB-IRIS was defined as new, recurrent or worsening respiratory symptoms and/or a worsening chest radiograph pulmonary infiltrate. Lymph node TB-IRIS was defined by enlarging peripheral lymph nodes on clinical examination, or enlarging thoracic nodes on chest radiograph or enlarging abdominal nodes on ultrasound or CT scan. Abdominal TB-IRIS was defined by abdominal symptoms (eg. abdominal pain or vomiting) attributed to TB-IRIS rather than an alternative cause (eg. adverse drug reaction), clinical hepatomegaly, or features of abdominal TB on ultrasound (eg. lymphadenopathy). Multi-organ TB-IRIS was defined by the presence of TB-IRIS symptoms, clinical signs or radiological/ultrasound abnormalities that involved more than 1 organ system (eg. pulmonary and nodal). These definitions relate to any features present at TB-IRIS presentation or that developed at any time during the course of the TB-IRIS episode. Only patients with abdominal symptoms had an abdominal ultrasound performed at time of TB-IRIS.

Prolonged paradoxical TB-IRIS was defined as TB-IRIS symptoms lasting more than 90 days. This was chosen because previous studies have reported a median duration of TB-IRIS of 1–3 months [5–11]. IRIS symptom onset date was as reported by the patient. IRIS end date was recorded as date of resolution of all symptoms attributed to TB-IRIS. If a patient was asymptomatic, but had only a persistent pulmonary infiltrate, tachycardia or hepatomegaly this did not constitute ongoing IRIS. In patients manifesting with lymph node enlargement due to TB-IRIS, IRIS resolution date was recorded when patients complained of no symptoms related to lymph nodes, and nodes had reduced in size by more than half of initial size or when node(s) were less than 2 cm in maximal diameter.

### Data collection

Clinical data regarding TB presentation and diagnosis, HIV history, TB-IRIS symptoms, corticosteroid treatment and duration, as well as laboratory results were extracted from the study databases using a standard data extraction form. When necessary the source notes were reviewed to extract other relevant data.

### Statistical analyses

Data analysis was performed using GraphPad Prism (La Jolla, CA) and R 3.0. Median and interquartile range (IQR) are presented for continuous variables and frequency and percentage for categorical variables. *P*-values were estimated from Fisher’s exact test (for categorical variables) and the Wilcoxon rank sum test (for continuous variables). *P*-values ≤ 0.05 were taken to be statistically significant.

**Fig. 1:**
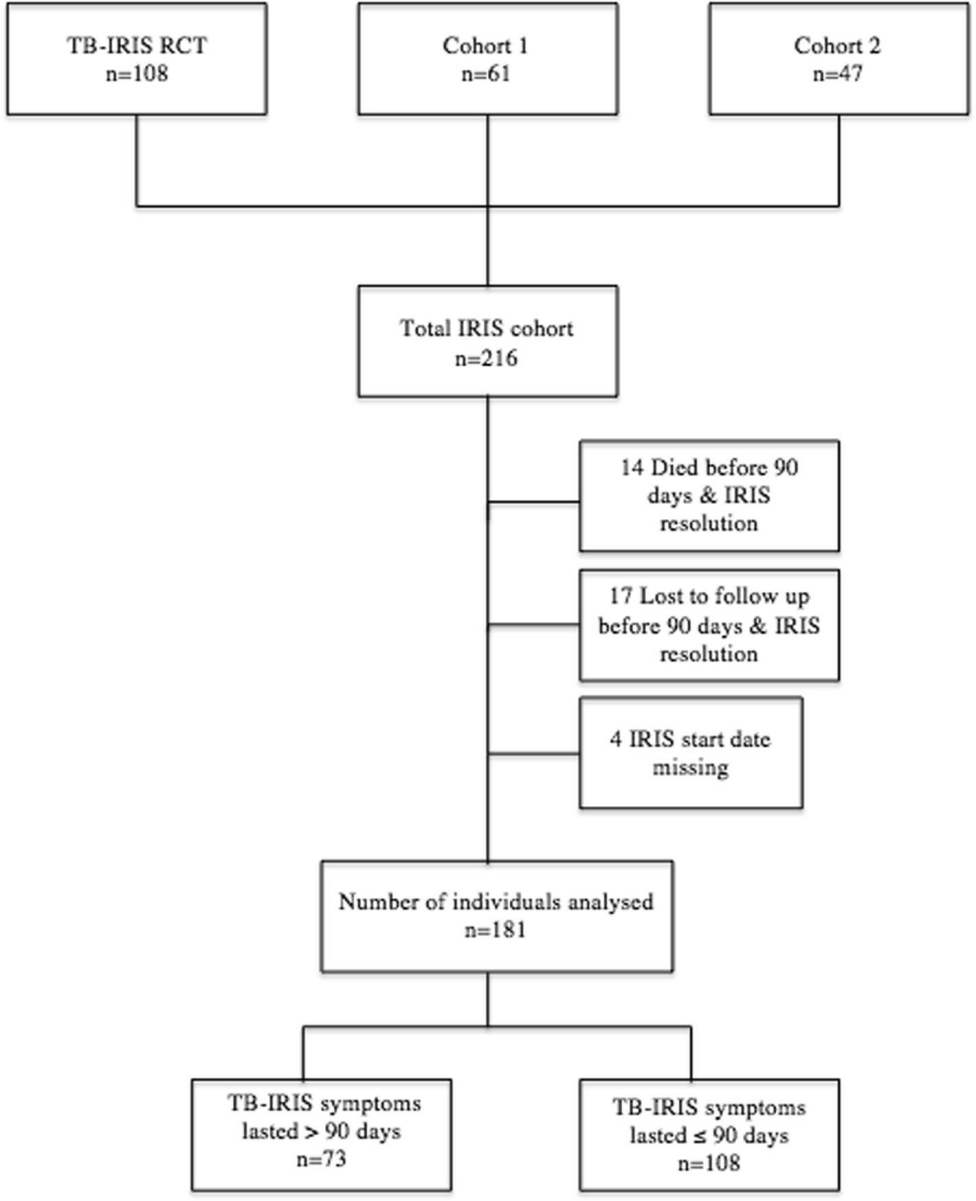
Inclusion and exclusion of patients in the prolonged TB-IRIS analyses. The total study population included 216 TB-IRIS patients from three prior studies (one randomised controlled trial and two prospective observational cohort studies). The initial analyses comparing those who experienced a prolonged course of TB-IRIS symptoms (defined as lasting more than 90 days) to those that did not experience a prolonged course of TB-IRIS included 181 patients. Reasons for exclusion of 35 patients from these analyses are shown. Abbreviations: ART = antiretroviral therapy, IRIS = immune reconstitution inflammatory syndrome, RCT = randomised controlled trial, TB = tuberculosis

The analyses were performed on a combined database from the 3 studies (Fig. 1). Duration of TB-IRIS symptoms and clinical features of those patients with prolonged TB-IRIS (>90 days and > 1 year) were described. Factors associated with prolonged TB-IRIS were analysed in univariate analysis, followed by multivariate logistic regression analysis. The multivariate logistic regression model included all variables with *p*-value < 0.1 by univariate analysis and certain pre-specified adjustment variables (eg. gender). A Cox proportions hazards model was developed in order to assess variables independently associated with time to resolution of TB-IRIS. Proportional hazards assumptions were verified using scaled Schoenfeld residuals. A competing risk analysis accounting for mortality was also performed.

For the analyses of factors associated with prolonged TB-IRIS (>90 days) patients who were lost to follow up or died prior to 90 days and before IRIS resolution were excluded from the univariate analyses and multivariate logistic regression models. They were included in the Cox proportional hazards and competing risks models and censored at death or loss to follow-up if these occurred before IRIS resolution.

## Results

Two-hundred and sixteen patients were diagnosed with paradoxical TB-IRIS across the three studies. One hundred eighty one patients were included in the initial analyses that compared patients with IRIS duration ≤ 90 days versus > 90 days. Reasons for exclusion from these analyses were mainly because patients were lost to follow-up or died before 90 days and before IRIS resolution (Fig. 1). In the Cox proportional hazards and competing risks models 212 patients were included, as these analyses allowed inclusion of patients who did not have a known IRIS resolution date (in four patients the TB-IRIS start date was not known and they were excluded). The characteristics of the 212 patients are summarized in Table 1.

**Table 1:** Baseline and TB-IRIS characteristics of the 212 patients included in Cox proportional hazards model

Variable	Median (interquartile range) or number (%)
Gender (*n* = 212)	
Female	138 (65.1 %)
Male	74 (34.9 %)
Age (years) (*n* = 212)	31 (27, 36)
WHO stage (*n* = 212)	
3	71 (33.5 %)
4	141 (66.5 %)
CD4 count prior to ART (cells/μl) (*n* = 199)	52 (28, 92.5)
CD4 count at TB-IRIS (cells/μl) (*n* = 95)	116 (71, 209.5)
Previous TB (*n* = 211)	55 (25.9 %)
Drug-resistant TB (*n* = 212)^a^	21 (10 %)
Initial TB diagnosis with extra-pulmonary involvement (*n* = 212)	134 (63.2 %)
Initial TB diagnosis with lymph node involvement (*n* = 212)	50 (23.6 %)
Duration from TB treatment to ART (days) (*n* = 211)	56 (31, 83)
Duration from ART to TB-IRIS onset (days) (*n* = 211)	14 (7, 21)
Hospitalised at time of TB-IRIS diagnosis (*n* = 206)	107 (50.5 %)
TB-IRIS lymph node involvement (*n* = 212)	80 (37.7 %)
TB-IRIS pulmonary involvement (*n* = 212)	83 (39.2 %)
TB-IRIS meningitis (*n* = 209)	10 (4.7 %)
TB-IRIS abdominal involvement (*n* = 212)	154 (72.6 %)
TB-IRIS multisystem involvement (*n* = 212)	166 (78.3 %)
Pulse rate (*n* = 134)	120 (107.3, 132)
C-reactive protein (mg/l) (*n* = 195)	103 (63, 158.5)
Haemoglobin (g/dl) (*n* = 136)	9.1 (8, 10.4)
White cell count (× 10^9^/l) (*n* = 132)	5.7 (4.1, 8.1)
ALT (IU/l) (*n* = 180)	34 (22, 51)
Alkaline phosphatase (IU/l) (*n* = 164)	159 (107, 274.8)
Corticosteroid treatment for TB-IRIS	125 (59.0 %)

Pulse rate and laboratory values refer to the value obtained when the patient was first assessed with TB-IRIS symptoms, unless otherwise stated. Numbers in brackets after the variable name refer to the number of patients for whom that variable was available

*Abbreviations*: *ALT* alanine transferase, *ART* antiretroviral therapy, *IRIS* immune reconstitution inflammatory syndrome, *TB* tuberculosis, *WHO* World Health Organisation

^a^Among 21 patients with drug resistant TB: four had rifampicin mono-resistance, three had isoniazid mono-resistance and 14 had resistance to rifampicin and isoniazid

Among the 181 participants included in the initial analyses, 116 (64.1 %) were female and their median age was 31 years (IQR 27–36). The median CD4 count prior to ART was 53 cells/μl (IQR 29–94) and at first TB-IRIS assessment was 117 cells/μl (IQR 71–218). The TB diagnosis was made by culture of *Mycobacterium tuberculosis* in 97 (53.6 %), positive smear for acid-fast bacilli in 41 (22.7 %) and clinical/radiological diagnosis in 43 (23.8 %). One-hundred and fifteen patients (63.5 %) had extra-pulmonary involvement at TB diagnosis. The median duration from starting TB treatment to ART was 56.0 days (IQR 31.0–81.3). Median duration from ART to TB-IRIS symptom onset was 13.5 days (IQR 7.0–19.3 days). The most frequent organ systems involved by TB-IRIS were: abdominal (*n* = 140, 77.3 %), pulmonary (*n* = 72, 39.8 %) and lymph nodes (*n* = 71, 39.2 %). In 150 patients (82.9 %) there was multi-system involvement. At the time of TB-IRIS diagnosis among the 181 patients, 83 (45.9 %) were hospitalised including all 47 patients in Cohort 2 (for five patients this data was missing).

The median duration of TB-IRIS symptoms was 71.0 days (IQR 41.0–113.2) for those who had a known TB-IRIS start and resolution date (*n* = 172, this excluded all those who died or were lost to follow-up prior to TB-IRIS resolution even if beyond 90 days). In the 181 patients, IRIS duration was longer than 90 days in 73 (40.3 %) and 6 (3.3 %) had IRIS duration of over one year. The distribution of duration is shown in Additional file 1: Figure S1.

One hundred and eleven of the 181 patients included in initial analyses (61.3 %) received prednisone treatment for TB-IRIS for a median 42 days (IQR 28–91). This included prednisone received as study drug or open-label prednisone in the RCT and as TB-IRIS treatment in the observational cohorts. In the RCT and in clinical practice standard practice was to start prednisone at a dose of 1.5 mg/kg/day. Outside of the RCT, clinician practice was to taper prednisone according to symptom response.

Among all 216 patients, 16 patients (7.4 %, 95 % CI 4.5 – 11.8 %) were known to have died a median of 33.5 days (IQR 28–61.5) after TB-IRIS onset. Fourteen patients died within 90 days and two patients died after 90 days. TB-IRIS was considered the main cause of death in five of the 16 deaths. The most frequent other cause of death among the TB-IRIS cases was sepsis (*n* = 5).

### Characteristics of patients with prolonged TB-IRIS

Among the 73 patients with TB-IRIS lasting > 90 days the following features were present either at presentation or at some time during the course of their TB-IRIS: lymph node involvement in 40/73 (54.8 %), pulmonary involvement in 23/73 (31.5 %), abdominal involvement in 59/73 (80.8 %) and pleural, pericardial effusions or ascites in 18/73 (24.7 %). In 64/73 (87.7 %) there was multi-system involvement. There were two deaths among the patients with prolonged TB-IRIS (>90 days). The characteristics, treatment and clinical course of the six patients who had TB-IRIS symptom duration > 1 year are summarized in Table 2.

**Table 2:** Characteristics, treatment and clinical course for six patients in whom TB-IRIS duration was longer than 1 year

	Age at TB-IRIS onset (years)	Pre-ART CD4 count (cells/μl)	Initial TB diagnosis	Initial IRIS features	Duration of TB-IRIS (days)	Duration of steroid treatment (days)	Features that persisted > 1 year	Comments
1	35	78	Extra-pulmonary TB (nodal)	Nodal	746 (IRIS ongoing at last visit)	70	Cerebellar tuberculomas/abscesses	The initial TB-IRIS episode involved lymph nodes and resolved on prednisone then patient re-presented several months later with new neurological manifestations and multiple rim-enhancing lesions in the cerebellum. Brain abscess resection tissue cultured drug-susceptible MTB. Experienced recurrence of TB-IRIS symptoms on tapering of steroids and required re-escalation of steroid doses.
2	34	160	Extra-pulmonary TB (nodal and miliary)	Nodal	824 (IRIS ongoing at last visit)	98	Supraclavicular nodes and large cold abscess	Experienced recurrence of TB-IRIS symptoms on tapering of steroids and required re-escalation of steroid doses.
3	56	65	Extra-pulmonary TB (miliary)	Nodal	426	0	Cervical and submandibular nodes	-
4	26	44	Extra-pulmonary TB (abdominal)	Abdominal	462	168	Axillary and cervical nodes, abdominal wall cold abscesses, mastitis	Patient found to have rifampicin mono-resistant MTB (urine culture sent at diagnosis) at TB-IRIS diagnosis. She was commenced on prednisone after commencement of appropriate treatment for rifampicin mono-resistant TB. Experienced recurrence of TB-IRIS symptoms on tapering of steroids and required re-escalation of steroid doses.
5	36	39	Extra-pulmonary TB (nodal and abdominal)	Nodal	1362	308	Multiple suppurating cervical nodes, psoas abscesses and abdominal pus collections	Experienced recurrence of TB-IRIS symptoms on tapering of steroids and required re-escalation of steroid doses. Patient still had residual left iliac fossa mass on abdominal imaging at TB-IRIS resolution that was decreasing in size and was asymptomatic.
6	39	80	Extra-pulmonary TB (nodal)	Nodal	519	137	Lymph nodes and cold abscesses	-

*Abbreviations*: *IRIS* immune reconstitution inflammatory syndrome, *MTB Mycobacterium tuberculosis, TB* tuberculosis

### Factors associated with prolonged TB-IRIS

Comparisons between those with prolonged IRIS and those with IRIS duration ≤ 90 days are shown in Table 3 (*n* = 181). In univariate statistical comparisons the following variables were significantly associated with IRIS duration > 90 days: drug-resistant TB, lymph node involvement at initial TB diagnosis, lymph node TB-IRIS and not being hospitalised at the time of TB-IRIS diagnosis. Treatment with corticosteroids was not associated with prolonged IRIS.

**Table 3:** Factors associated with prolonged course of paradoxical TB-IRIS (defined as TB-IRIS symptoms lasting longer than 90 days) (*n* = 181)

	TB-IRIS symptoms > 90 days (*n* = 73) (40.3 %)	TB-IRIS symptoms ≤ 90 days (*n* = 108) (59.7 %)	*p*-value for comparison
Female gender	50 (68.5)	66 (61.1)	
Male gender	23 (31.5)	42 (38.9)	0.39
Age (years)	31 (26, 35)	31 (27, 37)	0.32
WHO stage (*n* = 181)			
3	22 (30.1)	40 (37)	
4	51 (69.9)	68 (63)	0.42
CD4 count prior to ART (cells/μl) (*n* = 170)	57.5 (29, 91.2)	52 (29.2, 101)	0.77
CD4 count at TB-IRIS (cells/μl) (*n* = 89)	117 (71.7, 193.5)	116 (69, 262.5)	0.77
Previous TB (*n* = 180)	15 (20.6)	30 (28)	0.34
Drug-resistant TB (*n* = 181)^a^	10 (13.7)	4 (3.7)	0.02
Initial TB diagnosis with extra-pulmonary features (*n* = 181)	49 (67.1)	66 (61.1)	0.5
Initial TB diagnosis with lymph node involvement (*n* = 178)	25 (34.2)	19 (18.1)	0.02
Duration from TB treatment to ART (days)(*n* = 181)	58 (33, 84)	56 (31, 79.5)	0.8
Duration from ART to TB-IRIS onset (days) (*n* = 181)	13 (7, 23)	14 (7, 17)	0.51
Hospitalised at time of TB-IRIS diagnosis (*n* = 176)	25 (34.2)	58 (56.3)	0.01
TB-IRIS lymph node involvement (*n* = 181)	40 (54.8)	31 (28.7)	< 0.0001
TB-IRIS pulmonary involvement (*n* = 181)	23 (31.5)	49 (45.4)	0.07
TB-IRIS meningitis (*n* = 181)	1 (1.4)	5 (4.7)	0.42
TB-IRIS abdominal involvement (*n* = 181)	59 (80.8)	81 (75)	0.37
TB-IRIS multisystem involvement (*n* = 181)	64 (87.7)	86 (79.6)	0.17
Pulse rate (*n* = 120)	121 (105, 133)	119 (108, 130)	0.96
C-reactive protein (mg/l) (*n* = 172)	111 (74, 157.3)	95.5 (46.6, 165.7)	0.14
Haemoglobin (g/dl) (*n* = 113)	9.0 (8.0, 10.1)	9.1 (8.0, 10.6)	0.62
White cell count (×10^9^/l) (*n* = 109)	5.9 (4.2, 7.2)	6.0 (4.4, 8.6)	0.33
ALT (IU/l) (*n* = 157)	40 (25, 60)	35 (22, 48)	0.13
Alkaline phosphatase (IU/l) (*n* = 147)	164 (114, 261)	164 (95, 278)	0.46
Corticosteroid treatment for TB-IRIS	48 (65.8 %)	63 (58.3 %)	0.35

Medians (interquartile range) or number (%) are shown. Categorical variables were compared using Fisher’s exact test and continuous variables using Wilcoxon rank sum test. Pulse rate and laboratory values refer to the value obtained when the patient was first assessed with TB-IRIS symptoms, unless otherwise stated. Numbers in brackets after the variable name refer to the number of patients for whom that variable was available

*Abbreviations*: *ALT* alanine transferase, *ART* antiretroviral therapy, *IRIS* immune reconstitution inflammatory syndrome, *TB* tuberculosis, *WHO* World Health Organisation

^a^Among 14 patients with drug resistant TB: four had rifampicin mono-resistance, two had isoniazid mono-resistance and eight had resistance to rifampicin and isoniazid

In univariate logistic regression analyses (Table 4; unadjusted results) the following variables were significantly associated with IRIS duration > 90 days: lymph node involvement at initial TB diagnosis, drug-resistant TB and lymph node TB-IRIS. Hospitalisation at the time of TB-IRIS diagnosis was significantly associated with TB-IRIS resolving before 90 days as was the variable cohort. Hospitalisation and cohort were collinear and hence not included together in the same multivariate model. In our multivariate logistic regression model (Table 4; adjusted results) lymph node enlargement at time of TB-IRIS demonstrated a statistically significant association with IRIS duration > 90 days. Hospitalisation was associated with an IRIS duration ≤ 90 days. Drug-resistant TB demonstrated a borderline statistically significant positive association with prolonged TB-IRIS (aOR 3.26, 95 % CI 0.97–12.99).

**Table 4:** Odds ratios for univariate (unadjusted) and multivariate (adjusted) logistic regression models predicting the development of prolonged TB-IRIS (*n* = 181)

	Unadjusted OR (95 % CI)	Unadjusted *p*-value	Adjusted OR (95 % CI)	Adjusted *p*-value
Age (per 1 year increase)	0.97 (0.93–1.01)	0.16	0.98 (0.94–1.05)	0.48
Male gender	0.72 (0.38–1.35)	0.31	0.80 (0.39–1.63)	0.54
Lymph node involvement at initial TB diagnosis	2.36 (1.18–4.76)	0.02	1.79 (0.81–3.98)	0.15
Drug-resistant TB^a^	4.13 (1.32–15.56)	0.02	3.26 (0.97–12.99)	0.07
Hospitalised at time of TB-IRIS diagnosis	0.40 (0.22–0.75)	0.004	0.5 (0.25–0.99)	0.05
TB-IRIS lymph node involvement	3.01 (1.63–5.65)	0.0005	2.27 (1.13–4.59)	0.02
TB-IRIS pulmonary involvement	0.54 (0.29–1.02)	0.06	0.65 (0.32–1.30)	0.23
Cohort 2^b^	0.06 (0.01–0.18)	< 0.0001	Not included	
Cohort 1^b^	0.29 (0.12–0.62)	0.002	Not included	

*Abbreviations*: *IRIS* immune reconstitution inflammatory syndrome, *TB* tuberculosis

^a^The reference group is combined culture negative, culture not done and drug susceptible

^b^Reference cohort was randomized controlled trial

Due to the potential bias around Cohort 2 and hospitalisation (as all patients in Cohort 2 were admitted to a TB hospital from the time of starting ART and thus at TB-IRIS onset whereas in the other two studies patients were only admitted to a general hospital for shorter periods based on severity of illness at the time), a sensitivity analysis was performed excluding all individuals in Cohort 2. Both univariate and multivariate regression models were assessed (Additional file 2: Table S1) and although, as expected, the number of variables with statistically significant associations decreased, the direction and size of the effects remained largely the same. However, hospitalisation was no longer associated with prolonged TB-IRIS (aOR 1.16, 95 % CI 0.53–2.57, *p* = 0.71).

**Fig. 2:**
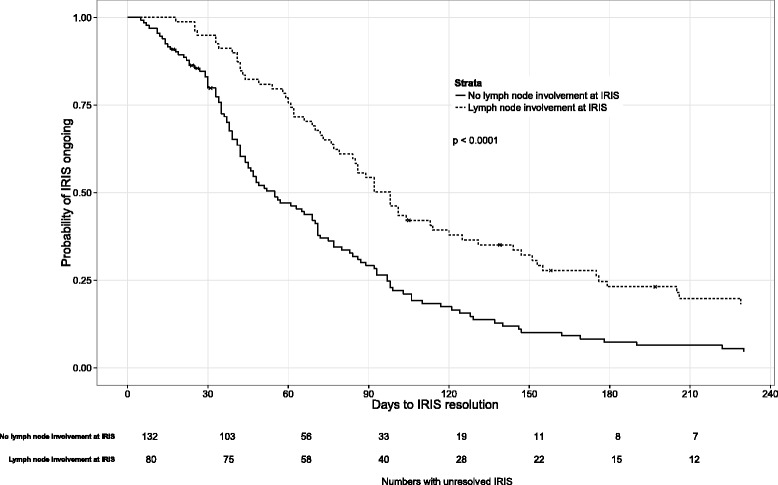
Kaplan-Meier plot showing time to TB-IRIS resolution. The graph shows the comparison of time to TB-IRIS resolution between patients who had TB-IRIS lymph node involvement to those patients who did not. Resolution was more rapid in those without lymph node involvement (*p* < 0.0001, log-rank test)

The Cox proportional hazards model (Table 5) included 212 patients for whom the TB-IRIS start date was known and a multivariate model was run based on predictors of interest as indicated by previous analyses and pre-specified variables. Predictors included were: lymph node involvement at initial TB diagnosis, TB-IRIS lymph node involvement, TB-IRIS pulmonary involvement, drug-resistant TB, as well as age, gender and hospitalisation. TB-IRIS lymph node involvement was statistically significantly associated with a lower hazard of TB-IRIS resolution (HR 0.55, 95 % CI 0.38–0.78; *p* = 0.0009) in both the full model and the model run without Cohort 2 (HR 0.60, 95 % CI 0.40–0.89; *p* = 0.01, Table 6). In a sensitivity analysis, the Cox proportional hazards model including patients from all three cohorts was re-run excluding patients with rifampicin resistance (*n* = 18) and excluding drug-resistance as a co-variate. TB-IRIS lymph node involvement remained significantly associated with outcome (data not shown). Additionally, a competing risks model was developed to account for the competing risks of death and IRIS resolution and the model for IRIS resolution demonstrated similar findings to that of the Cox proportional hazards model (Additional file 3: Table S2). In Fig. 2, the Kaplan-Meier plot demonstrates more rapid TB-IRIS resolution for those without lymph node TB-IRIS compared to those with TB-IRIS with lymph node involvement (*p* < 0.0001; log-rank test).

**Table 5:** Cox proportional hazards model predicting time to resolution of TB-IRIS symptoms

	Adjusted HR (95 % CI)	*p*-value
Age (per 1 year increase)	0.99 (0.97–1.01)	0.51
Male gender	1.22 (0.86–1.74)	0.27
Lymph node involvement at initial TB diagnosis	0.75 (0.50–1.15)	0.19
Drug-resistant TB	0.60 (0.34–1.08)	0.09
Hospitalised at time of TB-IRIS diagnosis	1.28 (0.92–1.78)	0.14
TB-IRIS pulmonary involvement	1.29 (0.92–1.81)	0.14
TB-IRIS lymph node involvement	0.55 (0.38–0.78)	0.0009

*Abbreviations*: *IRIS* immune reconstitution inflammatory syndrome, *TB* tuberculosis

**Table 6:** Cox proportional hazards model predicting time to resolution of TB-IRIS symptoms excluding Brooklyn Chest Hospital cohort (Cohort 2)

	Adjusted HR (95 % CI)	*p*-value
Age (per 1 year increase)	0.99 (0.96–1.01)	0.36
Male gender	1.42 (0.92–2.18)	0.11
Lymph node involvement at initial TB diagnosis	0.72 (0.44–1.17)	0.18
Drug-resistant TB	0.65 (0.36–1.20)	0.17
Hospitalised at time of TB-IRIS diagnosis	0.93 (0.62–1.40)	0.73
TB-IRIS pulmonary involvement	1.23 (0.82–1.83)	0.32
TB-IRIS lymph node involvement	0.60 (0.40–0.89)	0.01

In the Cox proportional hazards model, even when the Brooklyn Chest Hospital cohort (Cohort 2) was excluded, TB-IRIS lymph node involvement remained statistically significantly associated with a lower hazard of TB-IRIS resolution (aHR 0.60, 95 % CI 0.40–0.89; *p* = 0.01)

## Discussion

In this combined analysis that included patients enrolled in three previously reported studies the duration of paradoxical TB-IRIS symptoms was a median of 71 days (IQR 41–113), with 40.3 % of patients experiencing TB-IRIS symptoms lasting > 90 days. Six patients (3.3 %) experienced a very protracted course of TB-IRIS with symptoms > 1 year duration. TB-IRIS lymph node involvement was independently associated with a longer duration of symptoms.

The TB-IRIS case fatality rate in the combined cohort was 7.4 %, with 5 of the 16 deaths attributed to TB-IRIS. This is similar to the all-cause mortality rate reported in a recent TB-IRIS meta-analysis (7 %) [4], but lower than that reported in certain studies. In the CAMELIA trial mortality amongst the 155 TB-IRIS cases was higher than in our study (23 deaths among the 155 patients (14.8 %) with 6 deaths attributed to TB-IRIS), likely a consequence of the advanced immunosuppression at ART initiation in the CAMELIA participants (median CD4 = 27 cells/μl) [7].

Hospitalisation at TB-IRIS diagnosis was associated with IRIS duration ≤ 90 days (aOR 0.5, 95 % CI 0.25–0.99; *p* = 0.05). One possible explanation for these findings is that in Cohort 2 (study conducted at Brooklyn Chest TB Hospital) all patients were in hospital at time of ART initiation and there was close prospective follow up during early ART, TB-IRIS was likely diagnosed early and treated promptly with corticosteroids when needed thereby potentially reducing duration. This close follow-up may also have resulted in diagnosis of milder cases of inherently shorter duration. When Cohort 2 was excluded from a sensitivity analysis the association of prolonged TB-IRIS duration with hospitalisation was no longer observed.

Abdominal manifestations were the most frequent IRIS clinical feature in our study, which has not been reported in previous studies [4]. This was in part related to the permissive definition we used for abdominal IRIS which included any patient who had TB-IRIS fulfilling INSHI criteria and who in addition had an abdominal symptom attributed to TB-IRIS, hepatomegaly on clinical examination or abdominal ultrasound features of TB at the time of TB-IRIS. In a previous report, that included patients in this study, we reported over 50 % of TB-IRIS patients had hepatomegaly and many had cholestatic liver function derangement at TB-IRIS presentation [13].

In the 6 patients who had TB-IRIS lasting > 1 year the prolonged features were nodal enlargement and nodal suppuration as well as abscess formation, including cerebellar abscesses in one case. These patients received prolonged corticosteroids and four had one or more relapses when steroids were tapered or interrupted. However, in all of them corticosteroids were eventually stopped long before IRIS resolution because of the clinical impression that they were no longer providing benefit and concerns regarding cumulative toxicity. The longest duration of corticosteroid therapy was 308 days, and the longest duration of TB-IRIS was almost 4 years.

Prolonged IRIS represents a key management challenge in ART programmes. Prolonged IRIS has best been described in patients with *Mycobacterium avium* complex (MAC) and other non-tuberculous mycobacterial (NTM) infections. Phillips [17] reported 51 patients with NTM IRIS in whom the median duration of IRIS symptoms was 6 months with a range of 0–27 months. Riddell [18] reported the long-term outcomes of MAC-IRIS for 20 patients: 16 responded to treatment and were disease free after a mean of 17.4 months of therapy for MAC-IRIS, whereas four patients had persistent or relapsing disease despite 27 months of treatment. For paradoxical TB-IRIS several studies have reported on the duration of symptoms and cases with a prolonged course have previously been described. The median duration of TB-IRIS symptoms reported across studies has been between 40 and 90 days [5–9, 11]. In the SAPiT trial those patients who started ART within 4 weeks of TB treatment and developed TB-IRIS had a significantly longer duration of TB-IRIS (median 71 days) compared to TB-IRIS cases who started ART 8–12 weeks after TB treatment (median 34 days) [10]. In the CAMELIA trial, 155 cases of TB-IRIS were diagnosed, 77 % with nodal involvement and 38 % were treated with corticosteroids and 36 % with non-steroidal anti-inflammatory drugs. The median duration of IRIS was 7.4 weeks (IQR 4–19.8) [7]. Ollala et al. [11] reported a very variable duration of mycobacterial IRIS (between 19 days and more than 395 days, median of 57 days), and duration was longer in those patients whose paradoxical responses manifested the appearance or increase in lymphadenopathy (median 195 days) similar to our study. Huyst et al. [19] reported a case with two episodes of IRIS associated with TB; the first episode occurring soon after initiation of ART and the second episode occurring almost 4 years later. Similar to our findings, Burman et al. reported 10/25 (40 %) patients with TB-IRIS with a duration > 90 days. In two patients the duration of TB-IRIS was > 1 year and one required 108 aspirations of the IRIS lymph nodes [5]. Breton et al. reported four among 34 patients who had long term relapses lasting 14 months, 20 months, 3 and 4 years [12]. Naidoo et al. reported two cases of TB-IRIS unresolved after 18 months of trial follow-up in the SAPiT trial [10] and Michailidis et al. a case with a discharging psoas abscess for 15 months [9].

The risk factors most consistently implicated in the development of TB-IRIS are a low pre-ART CD4 count, short duration between starting TB treatment and ART, and extra-pulmonary TB [20]. Neither of the first two was found to be associated with prolonged TB-IRIS whereas TB-IRIS lymph node involvement (an extra-pulmonary manifestation) was, suggesting there is a common risk factor for developing TB-IRIS itself and for a prolonged course of TB-IRIS. In the CAMELIA trial mediastinal adenopathy was a risk factor for TB-IRIS [7] and in a Brazilian observational study superficial adenopathy was a TB-IRIS risk factor [21]. Nodal TB is also associated with a high incidence of paradoxical reactions in HIV-negative TB patients [22]. Multiple lines of evidence therefore suggest TB in a nodal location increases the risk for inflammatory reactions and in our study and the Ollala et al. study [11] a longer duration of these inflammatory reactions. In a recently published study Lerner et al. identified lymphatic endothelial cells (LECs), which line the lymphatic vessels in lymph nodes, as a niche for *Mycobacterium tuberculosis* replication. They suggest that these cells could represent an immune-privileged site [23]. The resultant abundant replication of *Mycobacterium tuberculosis* in these cells could result in high amounts of antigen in lymph nodes that become a stimulus for prolonged TB-IRIS.

A limitation of this study is that it included patients enrolled in three separate studies introducing heterogeneity. One of the studies was an RCT, and two were observational studies. The RCT and one of the observational studies (Cohort 1) were conducted at a referral hospital and the other observational study (Cohort 2) at an inpatient TB hospital. This weakness was mitigated by the use of the same diagnostic approach and case definition for TB-IRIS across the three studies. Also, the studies were all conducted by the same research group. Management protocols for TB-IRIS (apart from during the intervention period of the RCT) were similar across studies. Given that two of the studies were conducted at a referral hospital, this may have excluded milder cases of TB-IRIS who were not referred from primary care, biasing the findings towards patients with more severe TB-IRIS who may be more likely to die or have prolonged TB-IRIS. The location of the studies also affected the loss to follow-up reported: loss to follow-up was defined as loss to follow-up at the referral or TB hospital and did not necessarily mean the patient was also lost to follow-up at their primary care HIV or TB clinic. In the analyses we included multiple variables and no correction for multiple comparisons was conducted. Consistent with INSHI case definitions, the definitions of lymph node TB-IRIS and abdominal TB-IRIS were not mutually exclusive (ie. a patient with symptomatic enlarging abdominal lymphadenopathy was classified as having both nodal and abdominal TB-IRIS). Data relating to time of TB-IRIS onset relied on patient history which might have been subjective and prone to recall bias.

A strength of this study is the large number of patients with paradoxical TB-IRIS included. This is to our knowledge the largest number of TB-IRIS patients to be included in a single analysis, and this allowed us to present important and novel findings with respect to the clinical course of TB-IRIS and the contributing factors. All patients fulfilled the INSHI case definition, apart from the inclusion of certain patients with drug resistant TB who were also considered to have TB–IRIS.

## Conclusions

Around 40 % of patients with TB-IRIS have a clinical course of symptoms lasting longer than 90 days. Lymph node IRIS involvement is an independent risk factor for a prolonged course. In the small proportion of patients (3 %) with symptoms lasting more than 1 year this manifests with lymphadenitis and abscess formation. Whether earlier recognition and treatment of lymph node TB-IRIS could reduce the risk for prolonged TB-IRIS needs to be evaluated in future studies.

## Additional files

Additional file 1: Figure S1. Distribution of TB-IRIS duration. This graph shows the number and proportion of patients who had a duration of TB-IRIS symptoms within each of the specified duration categories (in days) displayed on the x-axis. The graph includes only the 172 patients who had a known TB-IRIS start and end date, and thus excludes 2 patients who had a duration >365 days but in whom IRIS was ongoing at last visit. (DOCX 174 kb) Additional file 2: Table S1. Odds ratios for univariate (unadjusted) and multivariate (adjusted) logistic regression models predicting the development of prolonged TB-IRIS excluding Brooklyn Chest Hospital cohort (Cohort 2). Footnote: Due to the potential bias around Cohort 2 and hospitalisation a sensitivity analysis was performed excluding all individuals in Cohort 2. Both univariate and multivariate regression models were assessed as shown in the table above and although, as expected, the number of variables with statistically significant associations decreased, the direction and size of the effects remained largely the same. However, hospitalisation was no longer associated with prolonged TB-IRIS (aOR 1.16, 95 % CI 0.53-2.57, *p* = 0.71). (DOCX 87 kb) Additional file 3: Table S2. Competing risks model of time to TB-IRIS resolution (with competing risk of death). Footnote: A competing risks model was developed to account for the competing risks of death and IRIS resolution. The model for TB-IRIS resolution demonstrated similar findings to the Cox proportional hazards model. (DOCX 73 kb)

## Abbreviations

aOR: Adjusted odds ratio
ART: Antiretroviral therapy
CAMELIA: CAMbodian Early versus Late Introduction of Antiretrovirals trial
CI: Confidence interval
DST: Drug susceptibility testing
HR: Hazards ratio
HREC: Human Research Ethics Committee
INSHI: International Network for the Study of HIV-associated IRIS
IQR: Interquartile range
IRIS: Immune reconstitution inflammatory syndrome
LECs: Lymphatic endothelial cells
MAC: *Mycobacterium avium* complex
NHLS: National Health Laboratory Services
NTM: Non-tuberculous mycobacteria
RCT: Randomized controlled trial
SAPiT: Starting Antiretroviral Therapy at Three Points in Tuberculosis trial
TB: Tuberculosis
TB-IRIS: Tuberculosis-associated immune reconstitution inflammatory syndrome
WHO: World Health Organization
