# Supplementary material for: Prolonged tuberculosis-associated immune reconstitution inflammatory syndrome: characteristics and risk factors
Source: BMC Infect Dis. 2016 Sep 27;16:518. doi: 10.1186/s12879-016-1850-2 (PMC5039896; doi:10.1186/s12879-016-1850-2)
Supplement: Additional file 1: Figure S1. — Distribution of TB-IRIS duration. This graph shows the number and proportion of patients who had a duration of TB-IRIS symptoms within each of the specified duration categories (in days) displayed on the x-axis. The graph includes only the 172 patients who had a known TB-IRIS start and end date, and thus excludes 2 patients who had a duration >365 days but in whom IRIS was ongoing at last visit. (DOCX 174 kb) [file 12879_2016_1850_MOESM1_ESM.docx]

**Additional file 1**


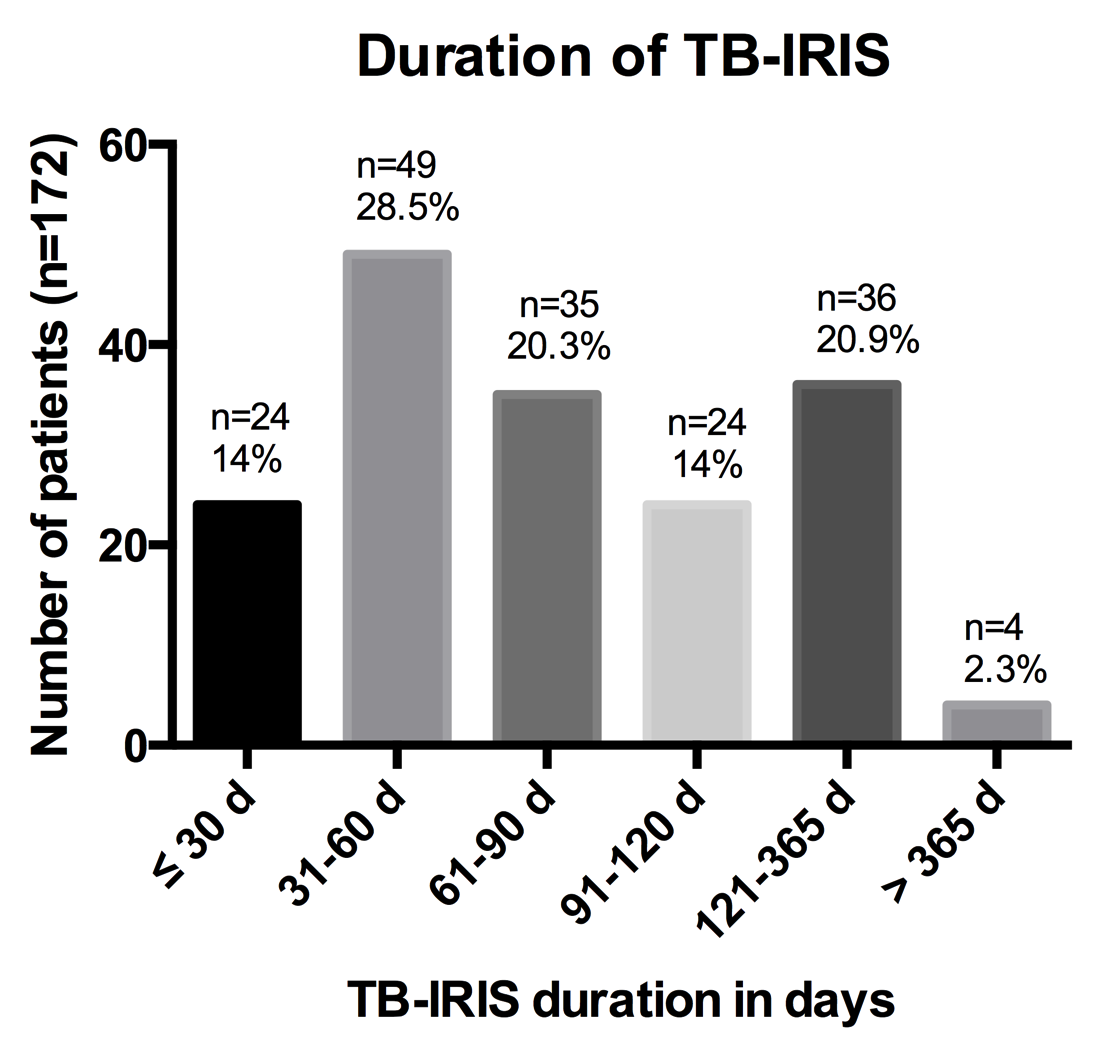


**Legend for Additional file 1**

**Additional file 1: Figure S1: Distribution of TB-IRIS duration**

This graph shows the number and proportion of patients who had a duration of TB-IRIS symptoms within each of the specified duration categories (in days) displayed on the x-axis. The graph includes only the 172 patients who had a known TB-IRIS start and end date, and thus excludes 2 patients who had a duration >365 days but in whom IRIS was ongoing at last visit.
