# Supplementary material for: Prolonged tuberculosis-associated immune reconstitution inflammatory syndrome: characteristics and risk factors
Source: BMC Infect Dis. 2016 Sep 27;16:518. doi: 10.1186/s12879-016-1850-2 (PMC5039896; doi:10.1186/s12879-016-1850-2)
Supplement: Additional file 2: Table S1. — Odds ratios for univariate (unadjusted) and multivariate (adjusted) logistic regression models predicting the development of prolonged TB-IRIS excluding Brooklyn Chest Hospital cohort (Cohort 2). Footnote: Due to the potential bias around Cohort 2 and hospitalisation a sensitivity analysis was performed excluding all individuals in Cohort 2. Both univariate and multivariate regression models were assessed as shown in the table above and although, as expected, the number of variables with statistically significant associations decreased, the direction and size of the effects remained largely the same. However, hospitalisation was no longer associated with prolonged TB-IRIS (aOR 1.16, 95 % CI 0.53-2.57, p = 0.71). (DOCX 87 kb) [file 12879_2016_1850_MOESM2_ESM.docx]

**Additional file 2: Table S1: Odds ratios for univariate (unadjusted) and multivariate (adjusted) logistic regression models predicting the development of prolonged TB-IRIS excluding Brooklyn Chest Hospital cohort (Cohort 2)**

|  | **Unadjusted OR (95% CI)** | **Unadjusted p-value** | **Adjusted OR (95% CI)** | **Adjusted p-value** |
| --- | --- | --- | --- | --- |
| Age  (per 1 year increase) | 0.98 (0.93, 1.03) | 0.36 | 0.99 (0.94, 1.05) | 0.723 |
| Male gender | 0.70 (0.34, 1.40) | 0.31 | 0.63 (0.28, 1.41) | 0.264 |
| Lymph node involvement at initial TB diagnosis | 2.36 (1.08, 5.39) | 0.03 | 1.79 (0.75, 4.46) | 0.20 |
| Drug-resistant TB | 2.79 (0.88, 10.60) | 0.10 | 2.31 (0.69, 9.11) | 0.19 |
| Hospitalised at time of TB-IRIS diagnosis | 0.98 (0.48, 2.03) | 0.96 | 1.16 (0.53, 2.57) | 0.71 |
| TB-IRIS lymph node involvement | 2.33 (1.19, 4.64) | 0.01 | 1.90 (0.89, 4.09) | 0.10 |
| TB-IRIS pulmonary involvement | 0.59 (0.29, 1.17) | 0.13 | 0.61 (0.28, 1.32) | 0.21 |

**Footnote:** Due to the potential bias around Cohort 2 and hospitalisation a sensitivity analysis was performed excluding all individuals in Cohort 2. Both univariate and multivariate regression models were assessed as shown in the table above and although, as expected, the number of variables with statistically significant associations decreased, the direction and size of the effects remained largely the same. However, hospitalisation was no longer associated with prolonged TB-IRIS (aOR 1.16, 95%CI 0.53-2.57, p=0.71).
