# Supplementary material for: Prolonged tuberculosis-associated immune reconstitution inflammatory syndrome: characteristics and risk factors
Source: BMC Infect Dis. 2016 Sep 27;16:518. doi: 10.1186/s12879-016-1850-2 (PMC5039896; doi:10.1186/s12879-016-1850-2)
Supplement: Additional file 3: Table S2. — Competing risks model of time to TB-IRIS resolution (with competing risk of death). Footnote: A competing risks model was developed to account for the competing risks of death and IRIS resolution. The model for TB-IRIS resolution demonstrated similar findings to the Cox proportional hazards model. (DOCX 73 kb) [file 12879_2016_1850_MOESM3_ESM.docx]

**Additional file 3: Table S2: Competing risks model of time to TB-IRIS resolution (with competing risk of death)**

|  | **Mortality** | | **TB-IRIS resolution** | |
| --- | --- | --- | --- | --- |
| **Characteristic** | **Adjusted HR (95% CI)** | **p-value** | **Adjusted HR**  **(95% CI)** | **p-value** |
| TB-IRIS pulmonary involvement | 0.81 (0.29-2.32) | 0.70 | 1.29 (0.92-1.81) | 0.13 |
| TB-IRIS lymph node involvement | 0.37 (0.11-1.18) | 0.09 | 0.55 (0.38-0.78) | 0.0009 |
| Lymph node involvement at initial TB diagnosis | 2.13 (0.72-6.26) | 0.17 | 0.75 (0.49-1.15) | 0.19 |
| Hospitalised at time of TB-IRIS diagnosis | 9.51 (2.05-44.04) | 0.004 | 1.28 (0.92-1.78) | 0.14 |
| Age (per 1 year increase) | 1.04 (0.99-1.10) | 0.14 | 0.99 (0.97-1.01) | 0.51 |
| Male gender | 0.32 (0.09-1.14) | 0.08 | 1.22 (0.86-1.74) | 0.27 |
| Drug-resistant TB | 1.96 (0.42-9.22) | 0.39 | 0.60 (0.34-1.08) | 0.09 |

**Footnote:** A competing risks model was developed to account for the competing risks of death and IRIS resolution. The model for TB-IRIS resolution demonstrated similar findings to the Cox proportional hazards model.
